# Dental Prophylaxis Decreases the Risk of Esophageal Cancer in Males; A Nationwide Population-Based Study in Taiwan

**DOI:** 10.1371/journal.pone.0109444

**Published:** 2014-10-03

**Authors:** Ya-Ling Lee, Hsiao-Yun Hu, Nan-Ping Yang, Pesus Chou, Dachen Chu

**Affiliations:** 1 Department of Dentistry, Taipei City Hospital, Taipei, Taiwan; 2 Institute of Public Health and Community Medicine Research Center, National Yang-Ming University, Taipei, Taiwan; 3 Department of Health Care Management, National Taipei University of Nursing and Health Sciences, Taipei, Taiwan; 4 Department of Neurosurgery, Taipei City Hospital, Taipei, Taiwan; 5 Department of Education and Research, Taipei City Hospital, Taipei, Taiwan; 6 Department of Education and Research, Keelung Hospital, Ministry of Health and Welfare, Keelung, Taiwan; University of Toronto, Canada

## Abstract

**Background:**

Periodontal disease (PD) is one of the most common chronic inflammatory diseases. Esophageal cancer (EC) is also a common cause of death due to cancer among males. Systemic inflammatory processes have been shown to increase the risk of cancer. We conducted a retrospective cohort study to investigate the association between PD and EC.

**Methods:**

A total of 718,409 subjects were recruited from the Taiwan National Health Insurance Research Database (NHIRD) and followed from January 1, 2000 to December 31, 2010. Of these, 519,831 subjects were diagnosed with PD and were grouped according to the most advanced treatment they received: dental prophylaxis, intensive treatment, or no treatment. The IRs of EC were compared among groups.

**Results:**

A total of 682 patients developed EC, resulting in an overall IR of 0.11 case-number per 1000 person-years (‰/y). The dental prophylaxis group had a significantly lower IR of EC (0.06‰/y) than other groups (*p*<0.001). Multivariable Cox regression analysis further revealed that male subjects [hazard ratio (HR) = 10.04, 95% confidence interval (CI)  = 7.58–13.30], as well as a history of esophageal ulcers (HR = 7.10, 95% CI = 5.03–10.01), alcohol abuse (HR = 5.46, 95% CI = 2.26–13.18), or esophageal reflux (HR = 1.86, 95% CI = 1.02–3.52), were factors associated with a higher risk of EC. And the dental prophylaxis group showed a significantly lower risk for EC (HR = 0.53, 95% CI = 0.44–0.65). Further subgroup analysis showed that the dental prophylaxis group among males had a significant lower risk (HR = 0.54, 95% CI = 0.44–0.66) for EC, while that of the females did not has statistically significant difference.

**Conclusion:**

For this cohort, subjects received dental prophylaxis reduced the risk of EC compared to all PD and no PD groups among males.

## Introduction

Periodontal disease (PD) is a chronic inflammatory disease of the gingiva and surrounding periodontal structures [1], [2]. Approximately 90% of the world’s population experiences mild to advanced PD [3]–[6]. PD is caused by specific bacterial biofilm, also known as dental plaque that accumulates around the teeth, and dental calculus (calcified plaque). This plaque can induce periodontal tissue inflammation, thereby damaging gingiva, periodontal connective tissue and the alveolar bone. If untreated, PD can eventually lead to tooth loss [7]–[9].

Esophageal cancer (EC) is ranked as the sixth most common cause of death due to cancer for males worldwide, and also in Taiwan [10], [11]. Furthermore, most of these cases are diagnosed in the late stages of EC, resulting in a poor prognosis [12]. Currently, the overall 5-year survival rate for patients with EC is less than 20% regardless of race or gender [13]–[15].

Increased inflammatory markers had been found among patients with PD, and activation of systemic inflammatory processes has been shown to increase the risk of cardiovascular disease, diabetes mellitus, premature low-weight birth, pulmonary disease [7], [16]–[19], and cancer, including EC [20]–[23]. Poor periodontal conditions and tooth loss are also associated with an increased risk of EC [23]–[28], although it remains unclear whether an association between PD and EC exists.

PD is a preventable and treatable disease. Moreover, dental prophylaxis has been reported to decrease the incidence of both stroke and cardiovascular diseases [19]. However, to the best of our knowledge, studies have not been conducted to assess the effects of PD treatment on the incidence of EC. Therefore, a large, population-based cohort study was conducted to estimate the risk of EC among different PD treatment groups over a 10-year follow-up period.

## Methods

### Data Sources

A compulsory, universal National Health Insurance (NHI) program has been established in Taiwan and it covers up to 99% of the nation’s inhabitants. The NHI research database (NHIRD) established by both the NHI Bureau and the National Health Research Institute (NHRI) provides useful epidemiological information for basic and clinical research in Taiwan. The NHRI administers the NHIRD in a manner that ensures all beneficiaries’ privacy and confidentiality, and provides access to researchers only upon ethical approval. Our study received full review by the Taipei City Hospital Institutional Review Board (NO: TCHIRB-1021110-E). The institutional review board has waived the need for written informed consent from study subjects because all potentially patient-identifying information was encrypted.

For the present study, the Longitudinal Health Insurance Database 2000 (LHID 2000) was used. The LHID 2000 is a standardized sample files for research use provided by NHRI, and consisted of comprehensive use and enrollment information for a randomly selected sample of 1 000 000 NHI beneficiaries, representing approximately 5% of all enrollees in Taiwan in 2000. All health care data of these subjects in the LHID 2000 were collected from January 1^st^ 2000 to December 31th 2010. A multistage stratified systematic sampling design was used and found that there were no statistically significant differences in sex or age between the sample group and all enrollees. Identification of each patient included in the LHID 2000 is encrypted, and each diagnosis is coded based on the *International Classification of Diseases, 9th Revision, Clinical Modification* (ICD-9-CM).

### Study Samples

**Figure 1:**
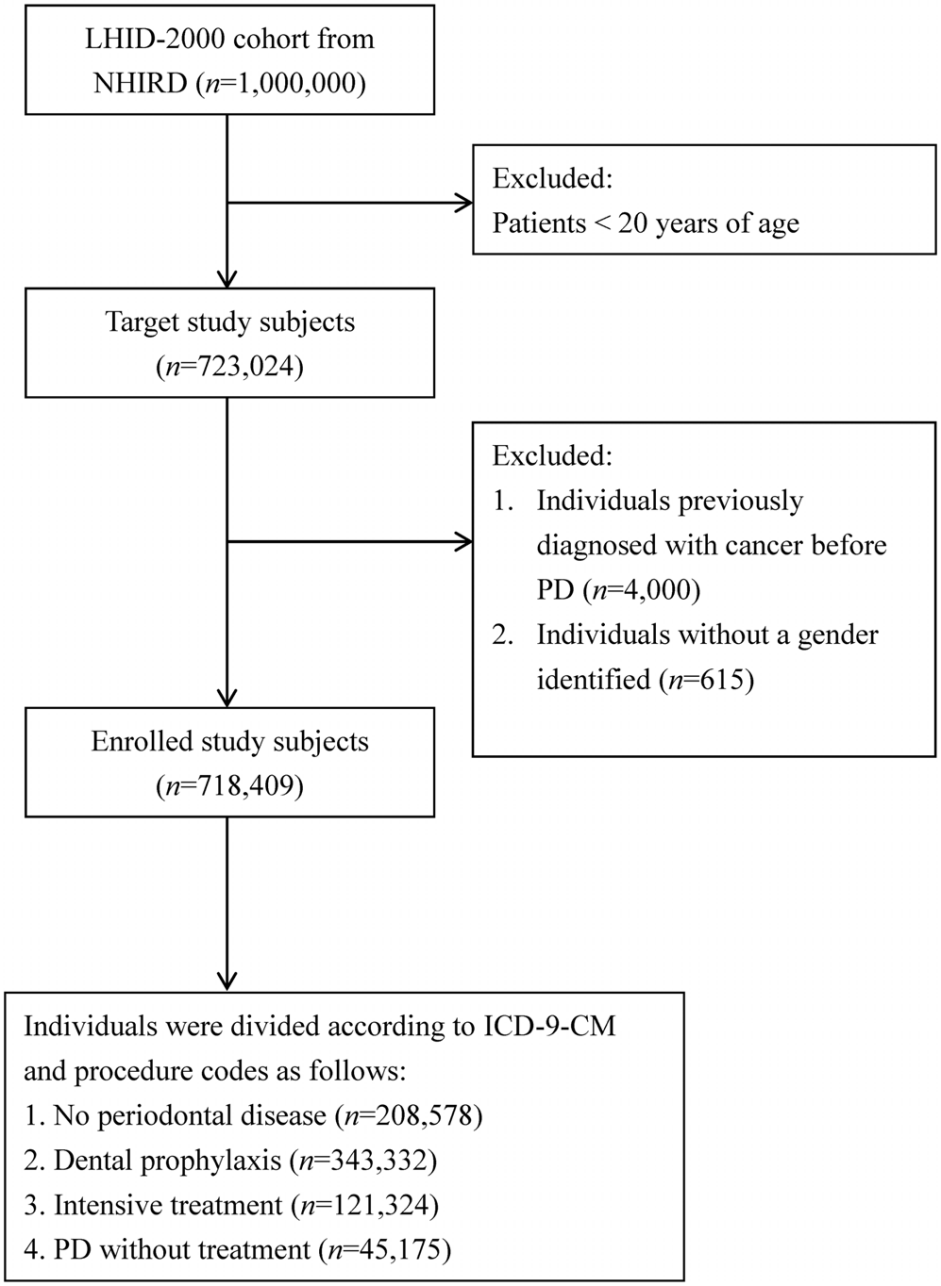
Selection of Study Patients.

The cohort for this retrospective study included 723,024 beneficiaries greater than or equal to 20 years of age (Fig. 1). The cohort entry date used for each PD subject was the date of the first ambulatory care visit between 2000 and 2010 that resulted in a diagnosis code for PD (ICD-9-CM: 523.0-523.5). The entry date for the control group (non- PD group) was the Jan 1, 2000. Individuals who had any cancer history before PD diagnosis (*n* = 4000), and individuals without an indicated gender (*n* = 615), were excluded from this study. Participants were followed from the entry date until the first date of hospitalization or outpatient visit due to EC (both ICD-9-CM 150 and Registry for catastrophic illness patients due to EC in NHIRD), death, or the end of the study period (31 December 2010).

Thus, data for 718,409 subjects monitored over 6,052,634 person-years were included in this study.

Patients with PD were divided into three groups according to the most advanced treatment that the patient received during the follow-up period: (1) dental prophylaxis group: PD patients who only received dental prophylaxis (*n* = 343,332; followed for 2,813,676 person-years); (2) intensive treatment group: PD patients who received intensive periodontal treatments, such as subgingival curettage and root planning or periodontal flap operation or tooth extraction (*n* = 121,324; followed for 795,327 person-years); and (3) PD without treatment group: PD patients who received no treatments (*n* = 45,175; followed for 171,622 person-years). Individuals that did not receive a diagnosis of PD during the study period served as the control group (*n* = 208,578; followed for 2,272,009 person-years).

An analysis of EC comorbidities and risk factors included diabetes mellitus (DM; ICD-9-CM 250), hypertension (HT; ICD-9-CM 401-405), hyperlipidemia (ICD-9-CM 272), esophageal ulcer (ICD-9-CM 530.2), Barrett’s esophagus (ICD-9-CM 530.85), alcohol abuse (ICD-9-CM 305.00-305.03), and esophageal reflux (ICD-9-CM 530.81; 530.11). Furthermore, only subjects with more than three outpatient visits during the study period were included.

### Statistical Analysis

Statistical analyses were performed using the SAS statistical package (version 9.2; SAS Institute, Cary, NC, USA). Patients were categorized into three groups according to age: 20–44 y, 45–64 y, and ≥65 y. Baseline characteristics for the entire cohort are presented in Table 1. The exposure was counted as time-dependent, and the incidence of EC among PD patients and controls was compared using the incidence rate (IR). The IR was calculated as case-number per 1000 person-years, (‰/y).

**Table 1:** Baseline characteristics of the study subjects.

Variables	n	EC	Follow-up person-year	ID	*p-value*
				(‰/yr)	
Total	718,409	682	6,052,634	0.11	
Gender					<0.001
Female	350,943	53	2,939,167	0.02	
Male	367,466	629	3,113,467	0.20	
Age at baseline (yr)					<0.001
20–44	435,539	166	3,620,914	0.05	
45–64	185,837	338	1,515,365	0.22	
≥65	97,033	178	916,355	0.19	
Periodontal disease					<0.001
No PD	208,578	358	2,272,009	0.16	
Dental prophylaxis	343,332	156	2,813,676	0.06	
Intensive treatment	121,324	134	795,327	0.17	
PD without treatment	45,175	34	171,622	0.20	
Co-morbidity					
Esophageal ulcer	4,563	36	37,190	0.97	<0.001
Alcohol abuse	805	5	6,472	0.77	0.001
Esophageal reflux	4,812	10	37,951	0.26	0.029
Diabetes mellitus	82,916	94	683,140	0.14	0.067
Hypertension	176,236	206	1,452,366	0.14	<0.001
Hyperlipidemia	115,745	93	922,864	0.10	0.079

The Cox proportional hazards model was used to calculate hazard ratios (HRs) and 95% confidence intervals (CIs). A Kaplan-Meier EC-free survival curve was generated using the Survival Analysis procedure in the STATA software to compare the cancer-free probability among the subgroups of PD [29], [30]. This model was adjusted for gender, patient age, and comorbidities (DM, HT, hyperlipidemia, esophageal ulcer, alcohol abuse, and esophageal reflux).

## Results

A total of 682/718,409 subjects developed EC between 2000 and 2010, resulting in an overall incidence rate of 0.11‰/y (Table 1). Males had a 10-fold higher EC incidence rate (EC-IR) compared to females (0.20‰/y vs. 0.02‰/y; *p*<0.001). The highest EC-IR was observed for subjects aged 45–64 y (0.22 ‰/y), followed by the 20–44 y group and the ≥65 y group (0.05‰/y and 0.19‰/y, respectively; *p*<0.001). Of the 208,578 no PD subjects, 358 developed EC (IR = 0.16‰/y). Univariable analysis revealed the highest EC-IRs were associated with a medical history of esophageal ulcers (0.97‰/y; *p*<0.001), followed by alcohol abuse (0.77‰/y; *p = *0.001), esophageal reflux (0.26‰/y; *p = *0.029), DM (0.14‰/y; *p* = 0.067), and HT (0.14‰/y; *p*<0.001).

Subjects who received dental prophylaxis had the lowest EC-IR (0.06‰/y) among no PD and PD groups. For the intensive PD treatment group, the EC-IR (0.17‰/y) was a little higher than that of the no PD group (0.16‰/y). In contrast, subjects with PD that did not receive any treatment had the highest EC-IR (0.20‰/y) compared with the other groups (*p*<0.001).

In a multivariable Cox regression analysis (Table 2), males were associated with a higher risk of EC than females (HR = 10.04, 95% CI = 7.58–13.30). In addition, subjects aged 45–54 y had the highest risk (HR = 4.90, 95% CI = 4.03–5.95), followed by subjects aged ≥65 y (HR = 3.56, 95% CI = 2.84–4.48) compared to subjects aged 20–44 y. Among all comorbidity subjects, individuals with esophageal ulcers exhibited the highest risk for EC (HR = 7.10, 95% CI = 5.03–10.01), followed by subjects with a history of alcohol abuse (HR = 5.46, 95% CI = 2.26–13.18) and then esophageal reflux (HR = 1.86, 95% CI = 1.02–3.52). In contrast, subjects with hyperlipidemia were associated with a lower risk value for EC (HR = 0.71, 95% CI = 0.56–0.91). Subjects with DM and HT did not exhibit a significant difference in their risk for EC compared to that without these comorbidities.

**Table 2:** Cox regression analysis to identify predictors of EC development.

Variables	Total		Male		Female	
	HR	95% CI	HR	95% CI	HR	95% CI
Gender						
Female	1.00					
Male	10.04	7.58–13.30				
Age						
20–44	1.00		1.00		1.00	
45–54	4.90	4.03–5.95	4.88	4.00–5.95	6.02	2.44–14.89
≥65	3.56	2.84–4.48	3.22	2.53–4.09	10.97	4.30–27.98
Periodontal disease						
No PD	1.00		1.00		1.00	
Dental prophylaxis	0.53	0.44–0.65	0.54	0.44–0.66	0.62	0.31–1.23
Intensive treatment	0.96	0.78–1.18	0.99	0.80–1.23	0.70	0.29–1.66
PD without treatment	1.27	0.89–1.82	1.38	0.96–1.98	0.37	0.05–2.78
Co-morbidity						
Esophageal ulcer	7.10	5.03–10.01	7.34	5.15–10.46	5.37	1.28–22.47
Alcohol abuse	5.46	2.26–13.18	5.49	2.27–13.25	-	-
Esophageal reflux	1.86	1.02–3.52	1.84	0.94–3.60	2.19	0.30–16.20
Diabetes mellitus	0.93	0.73–1.18	0.84	0.65–1.09	1.85	0.94–3.64
Hypertension	0.84	0.70–1.01	0.81	0.67–0.99	1.08	0.57–2.03
Hyperlipidemia	0.71	0.56–0.91	0.71	0.55–0.92	0.69	0.34–1.44

The dental prophylaxis group exhibited a significantly lower risk for EC (HR = 0.53, 95% CI = 0.44–0.65) than no PD and other PD groups after adjusting for gender, subject age, and comorbidities. Moreover, the intensive treatment group was associated with a lower risk (HR = 0.96, 95% CI = 0.78–1.18) and the PD without treatment group showed a higher risk (HR = 1.27, 95% CI = 0.89–1.82) for EC compared to the no PD group. However, both of the differences of the two groups were not statistically significant.

Further stratified study showed that the dental prophylaxis group among males was associated with a significant lower risk (HR = 0.54, 95% CI = 0.44–0.66) for EC while that of the female group did not has statistically significant difference.

**Figure 2:**
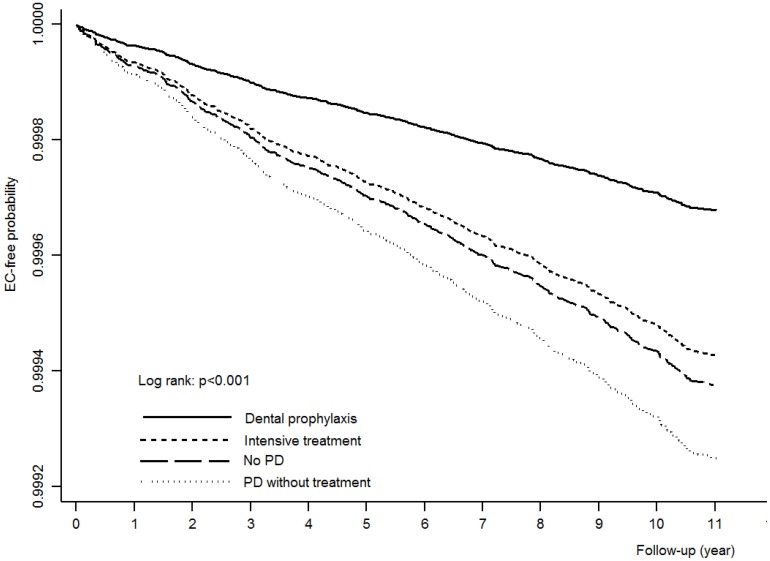
Kaplan-Meier EC-free probability curves for PD groups after adjusting for gender, patient age, and comorbidities.

Figure 2 shows the Kaplan-Meier EC-free survival curve among groups of the dental prophylaxis, intensive treatment, no PD and PD without treatment, during the time-period from 2000 to 2010 after adjusting for gender, subject age, and comorbidities. Subjects in the dental prophylaxis group were consistently associated with a higher cancer-free probability during the 10-year follow-up period analyzed compared with the other groups.

## Discussion

We explored the association of EC and PD using a population-based retrospective cohort study during a 10-year follow-up period. PD was found to be associated with an increased IR of EC, while dental prophylaxis was found to reduce the risk of EC among the males. These results support the theory that chronic inflammatory diseases such as PD play a role in the pathogenesis of EC. To our knowledge, this is also the first study to report the protective effect of dental prophylaxis for EC.

Edentulous individuals have almost a 2-fold higher risk of EC compared to subjects with at least 20 remaining teeth [24]. Moreover, two multicenter case-control studies reported that the loss of between 6 and 15 teeth resulted in a 2-fold greater risk of EC [25]. Tooth loss has also been associated with an elevated risk of EC [28]. Previous studies have also reported that PD is associated with an increased risk of oral and esophageal cancers [23]. In a case-control study by Sepehr et al., poor oral health was found to be a risk factor for premalignant esophageal squamous dysplasia [26].

The mechanisms responsible for the association between PD and cancer are not fully understood. According to one hypothesis, both the presence of carcinogenic metabolic by-products and an elevated inflammatory response are caused by PD [20], [23]. Moreover, up to 25% of human cancers have been reported to involve inflammation [31]. It has also been hypothesized that periodontal conditions reflect the systemic inflammatory status of an individual [6], [7]. For example, elevated expression of various inflammatory markers such as chemokines, cytokines, and prostaglandins may indirectly mediate systemic inflammation associated with PD [32]–[35]. The immune response induced by a chronic periodontal infection may also represent a cancer-causing factor [20], [23]. Conversely, PD may be prevented with good oral hygiene. For example, treatment of PD has been associated with lower serum concentrations of inflammatory markers [36], [37]. It has also been demonstrated that an increase in tooth-brushing frequency results in lower serum concentrations of CRP (C-reactive protein) and fibrinogen [38]. Moreover, individuals who brushed their teeth daily had a reduced risk of EC compared to individuals with poor oral hygiene [24], [28]. However, to our knowledge, the present study is the first to report the protective effect of professional dental prophylaxis for EC.

For the Taiwanese population studied, the EC-IR for males versus females was 0.2‰/y and 0.02‰/y, respectively. This higher incidence of EC in males is similar to that of the EC-IR for males and females reported for populations in Beijing (0.1‰/y and 0.04‰/y, respectively) and Osaka, Japan (0.1‰/y and 0.02‰/y, respectively) [27]. However, in Linxian, China, the EC-IR was as high as 1.0‰/y, and the male to female ratio was approximately 11. The lack of a statistically significant difference in the EC-IR for female subjects in the present study may be due to a lack of sufficient cases.

Esophageal ulcer and reflux esophagitis are associated with inflammation of the esophageal squamous epithelium and a significantly higher risk of EC [39]–[41]. Consistent with these observations, higher HRs were associated with these two diseases for the development of EC in the present study. Alcohol consumption is another strong risk factor for EC [42]–[44]. Correspondingly, a 4-fold higher risk for EC was identified for subjects with a history of alcohol abuse in the present study. In contrast, the association between DM and EC remains controversial. For example, while an elevated risk of EC has been identified for DM patients taking insulin or sulfonylurea [45], DM patients taking metformin did not exhibit an altered risk of EC [46]. In the present study, no statistically significant association between DM and EC was observed.

The strength of this study was the use of a nationwide population-based database that provided sufficient sample size and statistical power to assess the association between PD and EC. However, by using administrative data, a potential bias in diagnosis may have been introduced. Although, this may have been minimized since BNHI routinely samples patient charts from different medical centers in order to validate the quality of the database and to minimize miscoding or misclassifications. In addition, both ICD-9 diagnosis codes and PD treatment codes were used in the present study to define PD subjects. The EC patients were identified by both ICD-9 code and fulfill the criteria of registry for catastrophic illness patients due to EC in NHIRD. Furthermore, during the analysis of EC-IR for PD patients with other comorbidities, only those with more than three outpatient visits were included in order to minimize non-differential misclassification bias. Other misclassification bias may due to the nature of the PD. The severity of PD will change with the change of oral hygiene condition. And one may have had mild PD around some teeth and concurrently need intensive periodontal treatment in other area in the oral cavity. A clear distribution of PD subjects into few categories is difficult. However, we used the most extensive treatment that the patient received during the follow-up period to divide the subjects into 3 categories, so that to have a mutually exclusive classification and avoiding migration of patients among categories during the follow-up period. Another misclassification error may come from that some beneficiaries may never seek help from the dentist and may have diagnosed by untrained and not specifically calibrated dentists. However, Taiwan’s NHI is a compulsive system that has a 99% coverage rate and provided free dental prophylaxis twice a year. There is a very conscientious and careful dentist diploma and license system in Taiwan. These might reduce this error to a certain extend.

We adjusted some comorbidities or risk factors that may related to esophageal cancer. However, some of these comorbidities and risk factors may also be related to poor oral health. Adjusting these comorbidities and risk factors is necessary but may diminish the actual association between oral health and esophageal cancer. The lack of information regarding other PD and EC risk factors such as family history, body mass index, diet, and smoking status in the NHIRD may have reduced the feasibility and accuracy of interpreting the analytic outcomes. However, two previous case-control studies found that the association between poor oral health and EC was independent of smoking [25]. We included the risk factor of alcohol abuse by ICD-9-CM 305.00-305.03, as stated in the “Study sample”. However, the information source and the assessment of exposure to alcohol were not robust due to the limitation of a secondary database we used.

## Conclusion

In our study, male subjects who received dental prophylaxis showed a lower risk for EC compared to other PD and no PD groups. Further prospective studies should be carried out to evaluate the effect and possible mechanism of PD and dental prophylaxis on the development of EC.
